# Genome-wide identification and expression analysis of calmodulin and calmodulin-like genes in wheat (*Triticum aestivum* L.)

**DOI:** 10.1080/15592324.2021.2013646

**Published:** 2022-01-17

**Authors:** Yongwei Liu, Wenye Chen, Linbin Liu, Yuhuan Su, Yuan Li, Weizhe Jia, Bo Jiao, Jiao Wang, Fan Yang, Fushuang Dong, Jianfang Chai, He Zhao, Mengyu Lv, Yanyi Li, Shuo Zhou

**Affiliations:** a Institute of Biotechnology and Food Science, Hebei Academy of Agriculture and Forestry Sciences/Plant Genetic Engineering Center of Hebei Province, Shijiazhuang, China; b Hebei Seed Station, Shijiazhuang, China; c Handan Academy of Agricultural Sciences, Handan, China; d NCPC GeneTech Biotechnology Co. Ltd, Shijiazhuang, China

**Keywords:** Calmodulin, calmodulin-like protein, wheat, abiotice stress

## Abstract

Calmodulin (*CaM*) and calmodulin-like (*CML*) genes are widely involved in plant growth and development and mediating plant stress tolerance. However, the whole genome scale studies about *CaM* and *CML* gene families have not been done in wheat, and the possible functions of most wheat *CaM/CML* gene members are still unknown. In this study, a total of 18 *TaCaM* and 230 *TaCML* gene members were identified in wheat genome. Among these genes, 28 *TaCaM/CML* gene members have 74 duplicated copies, while 21 genes have 48 transcript variants, resulting in 321 putative *TaCaM/CML* transcripts totally. Phylogenetic tree analysis showed that they can be classified into 7 subfamilies. Similar gene structures and protein domains can be found in members of the same gene cluster. The *TaCaM/CML* genes were spread among all 21 chromosomes with unbalanced distributions, while most of the gene clusters contained 3 homoeologous genes located in the same homoeologous chromosome group. Synteny analysis showed that most of *TaCaM/CMLs* gene members can be found with 1–4 paralogous genes in *T. turgidum* and *Ae. Tauschii*. High numbers of *cis*-acting elements related to plant hormones and stress responses can be observed in the promoters of *TaCaM/CMLs*. The spatiotemporal expression patterns showed that most of the *TaCaM/TaCML* genes can be detected in at least one tissue. The expression levels of *TaCML17, 21, 30, 50, 59* and *75* in the root or shoot can be up-regulated by abiotic stresses, suggesting that *TaCML17, 21, 30, 50, 59* and *75* may be related with responses to abiotic stresses in wheat. The spatiotemporal expression patterns of *TaCaM/CML* genes indicated they may be involved widely in wheat growth and development. Our results provide important clues for exploring functions of *TaCaMs/CMLs* in growth and development as well as responses to abiotic stresses in wheat in the future.

## Introduction

As an important second messenger, calcium (Ca^2+^) plays pivotal roles in plant growth and development as well as stress signal transduction. Evidence shows that stimulations, such as plant hormones, gravity, light, cold, heat, drought, anoxia, salt, touch, wound and pathogen attack, can rapidly induce an increase of cytosolic-free Ca^2+^ concentrations ([Ca^2+^]_cyt_).^1^ These Ca^2+^ signatures can be decoded by effectors to generate specific responses.^2^ There are several Ca^2+^-sensing proteins found in plants, especially EF-hand domain containing proteins (i.e., a helix loop-helix structure), playing principal roles in Ca^2+^ signal transduction. The major EF-hand containing proteins could be divided into three families, including calmodulins (CaMs) and calmodulin-like proteins (CMLs), calcineurin B-like (CBL), and Ca^2+^-dependent protein kinases (CDPKs/CPKs).^3^

As a conservative Ca^2+^-binding protein, the typical CaM is a soluble protein composed of 149 amino acids that has two pairs of EF-hand domains, while each EF-hand domain can be combined with one Ca^2+^ ion.^4^ CaMs can interact with the target proteins and affect their biological activity to regulate the growth and development of plants and respond to stresses.^5^ CMLs share 16–75% amino acid identity with typical CaM, which usually carry 1–6 EF-hands and no other known functional domains with variable amino acids length 6. Most of the CaM/CMLs usually have no enzymatic or biochemical functions, except for CaM7, which can act as a transcription factor to directly regulate the expression of the *HY5* gene to regulate light morphogenesis.^7^ Different CaM/CMLs varied in the binding and regulation of target proteins [15], with slight differences in the structures of CaM/CML proteins that may result in considerable impacts on their binding to target proteins.^8^

CaM/CMLs are widely involved in plant growth and development and mediating plant stress tolerance.^5^ For example, the loss function of *AtCML24* and *AtCML25* can strongly affect pollen germination and pollen tube growth,^9,10^ and AtCML24 can interact with ATG4b to affect autophagy progression.^11^ *AtCML39* can be involved in regulating seed development, germination, fruit development, and early seedling establishment in *Arabidopsis*.^12,13^ It has been found that *CaM* is involved in heat shock signal transduction in wheat.^14^ AtCaM3 can regulate the activity of CaM-binding protein phosphatase (AtPP7) or protein kinase (AtCBK3) to increase the activation of the heat shock transcription factor (HSF), resulting in improving heat tolerance in *Arabidopsi*s.^15,16^ *ShCML44*, a *CML* gene isolated from cold tolerant wild tomato (*Solanum habrochaites*), can enhance the tolerance to cold, drought, and salinity stresses in plants, and promote germination and seedling growth.^17^ A rice calmodulin-like gene, *OsMSR2*, can enhance drought and salt tolerance and increase ABA sensitivity,^18,19^ while *OsCML4* can scavenge reactive oxygen species to improve drought tolerance in rice.^20^ The overexpression of *GmCaM4* can promote the DNA binding activity of the MYB2 transcription factor to increase salt tolerance in plants.^21^ AtCaM1 and AtCaM4 can be directly bind with S-nitrosoglutathione reductase (GSNOR), which can inhibit its activity and improve the internal level of nitric oxide (NO), resulting in increased salt resistance.^22^ In summary, itsuggests that *CaM/CMLs* play fundamental roles in Ca^2+^ signal transduction during development and stress adaptations in plants. However, the complex signal transduction of *CaM/CMLs* in regulating these pathways still needs to be explored.

Genes encoding *CaM/CMLs* have been identified at the whole genome scale in many plant species. For example, there are 7 *CaMs* and 50 *CMLs* in *Arabidopsi*s,^4^ 5 *CaMs* and 32 *CMLs* in rice (*Oryza sativa*),^23^ 6 *CaMs* and 52 *CMLs* in tomato (*Solanum lycopersicum*),^24,25^ 7 *CaMs* and 19 *CMLs* in lotus (*Lotus japonicas*),^26^ 79 *CMLs* in Chinese cabbage (*Brassica rapa* L. ssp. *pekinensis*),^27^ and 4 *CaMs* and 58 *CMLs* in apple (*Malus* × *domestica*).^28^ In wheat, the cDNAs corresponding to 10 *CaM* genes have been isolated, which can be classified into 4 subfamilies. Using subfamily-specific DNA probes, the southern-blot analysis showed that there may be 10–20 copies of *CaM* genes located in the wheat genome.^29^ However, up to now, there are only 2 *CML* genes that have been reported in wheat. Overexpression of *TaCML20* can enhance water soluble carbohydrate accumulation and yield in wheat,^30^ and *TaCML36* can positively participate in an immune response to *Rhizoctonia cerealis*.^28^ Due to its large and complex genome, the whole wheat genome sequencing was relatively later than other model plant species.^31^ There is no study about *CaM* and *CML* gene families of wheat in whole genome scale, and the possible functions of most wheat *CaM/CML* gene members are still unknown.

In this study, 18 deduced *CaM* and 230 *CML* gene members were identified in the wheat genome using BlastP and HMM-search methods, and their duplication copies, transcript variations, gene structure, protein motifs, isoelectric point, molecular weight, subcellular location, and subgroup classification were analyzed. We also analyzed the spatiotemporal expression patterns and expression profiles after abiotic treatments. This systematic analysis of the complete sets of *CaM/CMLs* in wheat will provide useful information for further gene cloning and functional exploration as well as the understanding of the Ca^2+^-mediated signal transductions in wheat.

## Materials and methods

### *Identification and characterization of* TaCaM/CML *genes in wheat*

The CaM/CML proteins were identified and characterized following the method as described by Wang et al with some modifications.^32^ Protein sequences of wheat (*Triticum aestivum*, IWGSC 1.1), *Triticum turgidum* (Svevo_v1), *Aegilops tauschii* (Aet_v4.0) were downloaded from the Ensemble database (http://plants.ensemble.org) to construct the local protein database. The database was then searched using known CaM and CML protein sequences collected from *A. thaliana* (7 CaMs and 50 CMLs), *O. sativa* (5 CaMs and 32 CMLs), and *T. aestivum* (10 CaMs) using the local BLASTP program with an e-value of 1e^−5^ and a threshold of 50% identity. Furthermore, a hidden Markov model (HMM) profile of EF-hand (PF00036) was downloaded from the Pfam database (http://pfam.xfam.org/) and used to search the wheat local proteins database using the HMM-search tool embedded in HMMER 3.2.^33,34^ BLAST and HMMER hits were compared and parsed by manual editing. Furthermore, a self blast of these sequences was performed to remove the redundancy, and the remaining sequences were considered as the putative TaCaM and TaCML proteins, These sequences were submitted to the NCBI Batch CD-search database (https://www.ncbi.nlm.nih.gov/Structure/bwrpsb/bwrpsb.cgi) to confirm the presence and integrity of the EF-hand domain and without any other domains. The theoretical pI (isoelectric point) and Mw (molecular weight) of the putative TaCaM and TaCML proteins were calculated using the compute pI/Mw tool online tool (http://web.expasy.org/compute_pi/).^35^ Subcellular localization of these genes was predicted using the CELLO v2.5 web server (http://cello.life.nctu.edu.tw/).^36^

### Multiple sequence alignments and phylogenetic analysis

Multiple sequence alignments were generated using the ClustalW tool.^37^ To investigate the evolutionary relationship among TaCaM/CML proteins, a maximum likelihood (ML) tree was constructed by MEGA X software based on the full-length of the TaCaM/CML protein sequences.^38^ Bootstrap test method was adopted, and the replicate was set to 1000.

### Gene structure and conserved motifs analysis

Gene structures of the *TaCaM/CML* members were predicted using the gene structure display server 2.0 (http://gsds.gao-lab.org/) using the genomic and coding sequences.^39^ The conserved motifs of TaCaM/CML proteins were predicted using the MEME program (http://meme-suite.org/tools/meme) with the following parameters: a maximum of 10 motifs and an optimum motif width between 10 and 50 amino acids.^40^

### *Chromosomal locations and synteny analysis of* TaCaM/CML *genes*

The chromosomal locations were collected from the Ensemble plant database (http://plants.ensembl.org) and the location map was constructed using the TBtools v0.6654 software.^41^ The Multiple Collinearity Scan toolkit (MCScanX) was used to analyze the gene duplication events.^42^

### *Prediction of* cis*-acting elements in the promoters of* TaCaM/CML *genes*

To investigate the *cis*–acting elements in the promoter sequences of the *TaCaM/CML* genes, 2-kb sequences upstream of the initiation codon (ATG) were collected from the Ensemble plant database (http://plants.ensembl.org) and subjected to the PLACE database (https://www.dna.affrc.go.jp/PLACE).^43^

### *The spatiotemporal expression patterns of* TaCaM/CML *genes*

The spatiotemporal expression patterns of *TaCaM/CML* genes were analyzed following the method as described by Tyagi et al. with some modifications.^44^ High-throughput RNA sequence data with two biological replicates (PRJEB5314) were used for the expression analysis of the *TaCaM/CML* genes in three developmental stages of five different tissues, including the root, stem, leaf, spike, and grain.^45^ The transcripts per million mapped (TPM) values were used for the calculation of the expression data at a *P*-value of 0.001. The correlation between the replicates was determined by using log_2_^TPM^-transformed values for the tissue developmental stages. Heatmaps were constructed using the TBtools v0.6654 software.^41^

### Plant Materials and Treatments

Plant growth and abiotic stress treatments were conducted as described previously.^46^ Jinhe 9123, a wheat cultivar bred by ourselves and preserved in our lab, was used in this study. The seeds were surface-sterilized for 10 minutes in 1% NaOCl and repeatedly rinsed with tap water three times, then seeded in 1/2 Hoagland nutrient solution after immersion and imbibition for 12 h, and hydroponically cultivated in the incubator with a 16/8-h photoperiod at 25°C. Four 10-day-old homogeneous seedlings groups, each of which included 100 seedlings, were subjected to different treatments, including 16.1% PEG6000, 200 mM NaCl, cold (4°C), and heat (40°C). The seedlings without any treatment were used as control. The root and shoot tissues from ten seedlings were sampled at one time point for the experiment. Collected samples were immediately frozen in liquid nitrogen and stored at −80°C for RNA extraction.

### RNA isolation and gene expression analysis

RNA isolation and gene expression analysis were carried out following the method as described by Yang et al. with some modifications.^46^ Total RNA of collected samples was isolated using the EasyPure® Plant RNA Kit (ER301-01, Transgen, China). For reverse transcription, the first-strand cDNA was synthesized using a PrimeScript^TM^ RT reagent kit (RR047A, TaKaRa, Japan). Quantitative real-time PCR (qRT-PCR) for examination of the *TaCMLs* expression patterns were performed using the TB Green^TM^ Premix Ex Taq^TM^ II (RR820A, TaKaRa, Japan) with 7500 Real-Time PCR System (Applied Biosystem, USA). Gene cluster-specific and internal reference gene *TaActin* primers were listed in additional file 4: Table S2. The qRT-PCR program was carried out as follows: pre-denaturation at 95°C for 30 s; denaturation at 95°C for 5 s, annealing at 58°C for 30 s, extension at 72°C for 34 s, 45 cycles. The 2^−ΔΔCt^ method was used to analyze the data.^47^ All of the experiments were performed with three technical replicates and three biological replicates, and the data were represented by mean value ± standard error of three biological replicates.

## Results

### *Identification and characterization of* TaCaM/CML *genes in wheat*

**Figure 1.**
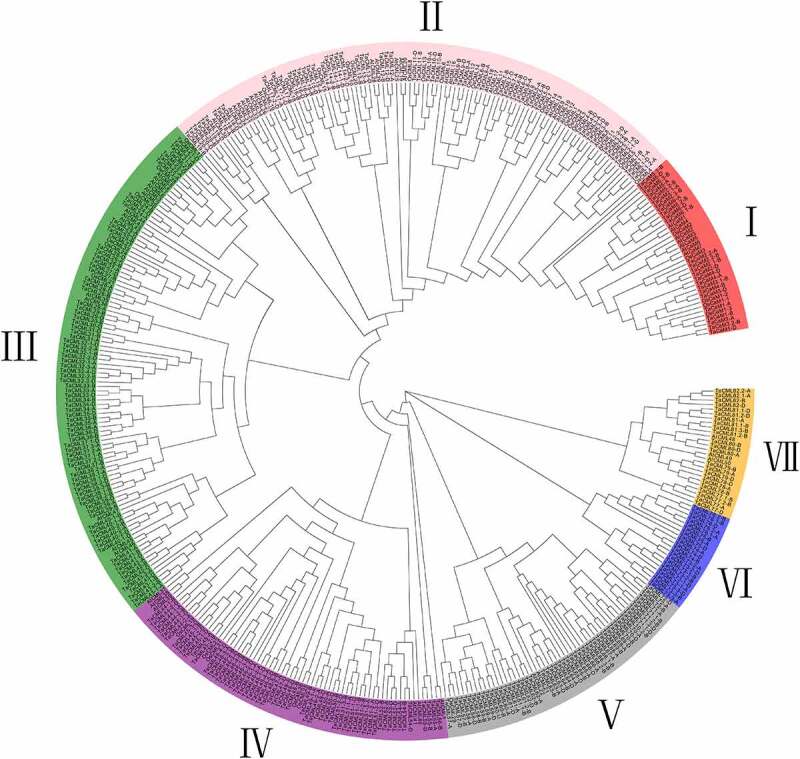
Phylogenetic relationship of CaM/CML proteins. The phylogenetic tree of CaM/CML proteins from wheat, *Arabidopsis*, and rice is shown in this figure. The phylogenetic tree was generated using the maximum likelihood (ML) method in MEGA X. A bootstrap test method was adopted with 1000 replicates.

Using BlastP and HMM-search methods, a total of 18 *TaCaM* and 230 *TaCML* gene members were identified in wheat genome. It can be found that 28 *TaCaM/CML* gene members have 74 duplicated copies, which located adjacently on the chromosome and from the same cluster in phylogenetic tree; as while 21 genes have 48 transcript variants, resulting in 321 putative TaCaM/CML proteins totally (Additional file 1: Table S1). To obtain the subfamily classification of the 321 TaCaM/CML proteins, multiple sequence alignment was performed using the amino acid sequences, and a maximum likelihood (ML) tree was constructed. The TaCaM/CML proteins were clustered into 7 groups according to their similarities and relationships with each other, and each group contained 18, 57, 65, 34, 44, 12, and 18 gene members, while the group I was *CaM* family. We also found that 18 *TaCaM* members were grouped into 6 gene clusters, and 230 *TaCML* gene members were grouped into 82 gene clusters (Figure 1). The predicted wheat *CaM/CML* genes were designated as *TaCaM1* to *TaCaM6* and *TaCML1* to *TaCML82*, plus the suffix corresponding to the specific wheat sub-genome identifier (A, B, or D). The genes in the same cluster were considered as the homoeologous genes of one wheat *CaM/CML* cluster. It showed that 77 *TaCaM/CML* gene clusters each contained 3 homoeologous gene members, while 6 other gene clusters (*TaCML4-B/D, 8-A/B, 11-B/D, 23-A/D, 30-B/D*, and *47-A/D*) harbored 2, and 5 remaining gene clusters (*TaCML22-A, 53-D, 56-B, 67-A*, and *68-A*) had 1 (Figure 1).

The genes located adjacently on the chromosome and from the same cluster in phylogenetic tree were difined as duplicated genes, such as TraesCS5A02G057100.1 and TraesCS5A02G057200.1 from *TaCML2-A* gene cluster. The duplicated genes were named as gene member plus duplication number and wheat sub-genome identifier. For example, duplicated genes of *TaCML2* member are named as *TaCML2-1-A* (TraesCS5A02G057100.1) and *TaCML2-2-A* (TraesCS5A02G057200.1). It showed that 28 gene members have 74 duplicated copies, including 16 genes (*TaCML2-A/D, 14-B/D, 17-D, 32-B, 40-A/B/D, 43-A/B, 44-A/B/D, 45-B*, and *49-A*) have2 duplicated copies, 8 genes (*TaCML32-A, 45-D, 51-A/B/D*, and *73-A/B/D*) have 3 duplicated copies, 2 genes (*TaCML32-D*, and *46-A*) have 4 duplicated copies, and 2 genes (*TaCML46-B/D*) have 5 duplicated copies (Additional file 1: Table S1).

The transcript variations are named as gene member plus transcript variation number and wheat sub-genome identifier. For example, transcript variations of *TaCaM2-A* cluster are named as *TaCaM2.1-A* and *TaCaM2.2-A*. It can be found that 21 genes have 48 transcript variants, including 16 gene members (*TaCaM2-A, 3-B*, and *5-B; TaCML1-A, 37-D, 38-A/D, 61-B, 71-B/D, 76-A/B, 77-B, 81-D*, and *82-A*) have 2 types of transcript variants, and 5 genes (*TaCaM2-B, 6-B*; and *TaCML37-A, 38-B, 66-B*, and *81-B*) have 3 types of transcript variants (Additional file 1: Table S1).

The TaCaM proteins ranged in length from 128 (TaCaM5.2-B) to 183 (TaCaM6.1-B), with molecular weights ranging from 14.50 kDa (TaCaM5.2-B) to 20.48 kDa (TaCaM6.1-B) and isoelectric points ranging from 3.83 (TaCaM1-B) to 4.25 (TaCaM6.2-B). These TaCML proteins ranged in length from 53 (TaCML46-3-B) to 1299 (TaCML81.3-B) amino acids, with molecular weights ranging from 5.65 kDa (TaCML46-3-B) to 139.62 kDa (TaCML81.3-B) and isoelectric points ranging from 3.70 (TaCML57-D) to 10.28 (TaCML33-B/D). Additionally, 289 TaCaM/CML proteins are acidic proteins, while only 32 TaCMLs (TaCML18-A/B/D, 32-1-A/B/D, 32-2-A/B/D, 32-3-A/D, 32-4-D, 33-A/B/D, 34-A/B/D, 36-A/B/D, 55-B, 61-A/D, 61.1/2-B, 66-A/D, 66.1/3-B, 67-A, and 68-A) were basic proteins with isoelectric points higher than 7. Subcellular localization prediction indicated that 253 TaCaM/CML proteins are localized in the cytoplasm, 20 in the nucleus, 13 in the chloroplast, 5 in the mitochondria, 15 in the extracellular matrix, 8 in the periplasm, 6 in the outermembrane, and 1 protein in the plasma membrane (Additional file 1: Table S1).

### Gene structure and conserved motifs of TaCaM/CMLs

The exon and intron structures of *TaCaM/CML* genes were analyzed using the GSDS database. The numbers of introns in all the *TaCaM/CML* gene transcripts varied from 0 to 15, in which 184 *TaCaM/CML* transcripts has no intron, as well as the transcripts of 26, 13, 17, 20, 19, 6, 8, 3, 4, 5, 3, 7, 4, 1 and 1 have 1, 2, 3, 4, 5, 6, 7, 8, 9, 10, 11, 12, 13, 14 and 15 introns, respectively (Additional file 1: Table S1; Additional file 2: Figure S1).

The conserved motifs of the TaCaM/CML proteins were predicted using the MEME program. A total of 10 motifs were identified among the 321 TaCaM/TaCML proteins. Motif 1 – the predicted EF-hand domain – was observed in all of the TaCaM/CML proteins, while motif 10 only appeared in 13 TaCML proteins of *TaCML37/38* clusters (TaCML37.1/2/3-A, 37-B, 37.1/2-D, 38.1/2-A, 38.1/2/3-B, and 38.1/2-D). It showed that 37, 90, 79, 109, and 6 TaCaM/CML proteins contain 1, 2, 3, 4, and 5 EF-hands, respectively. (Additional file 1: Table S1; Additional file 3: Figure S2). In general, genes and proteins belonging to the same cluster contained similar structures and motifs. Among 83 gene clusters harboring multiple-gene members, similar gene structures with the same numbers of exons and coding exons can be observed in 59 gene clusters, and similar distributions of protein motifs can be found in 56 gene clusters (Additional file 1: Table S1; Additional file 2: Figure S1; Additional file 3: Figure S2).

### *Chromosome locations and synteny analysis of* TaCaM/CML *genes*

**Figure 2.**
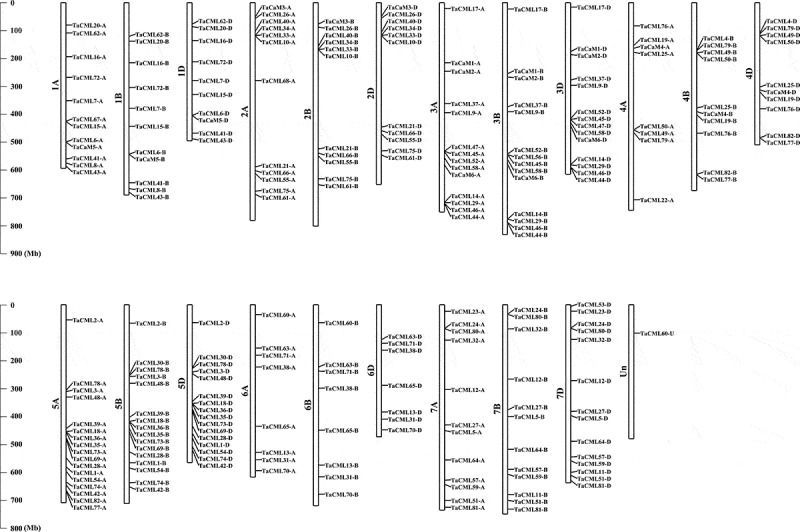
Chromosome locations of wheat *CaM/CML* genes. The chromosome numbers are shown at the left of each bar. The scale is represented in megabases (Mb).

To determine the distribution of *TaCaM/CML* genes in 21 chromosomes of wheat, we analyzed their chromosomal locations. Among 248 wheat *CaM*/*CML* genes, 83, 82, and 82 gene members were mapped in each of the sub-genomes A, B, and D, respecitively; while the *TaCML60-U* gene was located on the unanchored scaffolds. The *TaCaMs/CMLs* genes were spread among all 21 chromosomes with unbalanced distributions. 33, 34, 42, 28, 49, 23, and 38 *TaCaM/CML* genes were located in homoeologous-group 1, 2, 3, 4, 5, 6, and 7 chromosomes, respectively. Additionally, chromosome 5A had the highest numbers of 17 *TaCaM*/*CML* gene members, and chromosome 6D contained the lowest numbers of 7 gene members (Figure 2).

**Figure 3.**
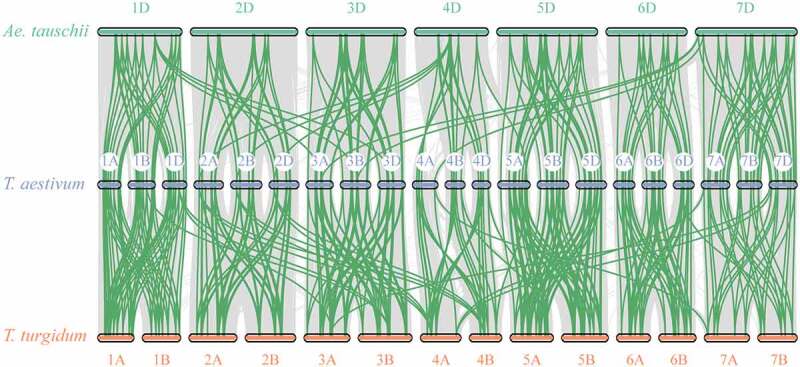
Collinearity diagrams among *TaCaM/CML* gene members of *T. aestivum, T. turgidum*, and *Ae. Tauschii*. Orthologous *CML* genes mapped onto *T. aestivum* (chromosomes 1A–7D), *T. turgidum* (chromosomes 1A-7B), and *Ae. Tauschii* (chromosomes 1D-7D). Green lines indicate orthologous *CaM/CML* genes pairs.

To understand the evolution of *TaCaM/CML*, the *CaM/CML* gene members were identified in ancestral species, *T. turgidum* (AABB) and *Ae. tauschii* (DD) genome. It showed that there were 152 and 88 *TaCaM/CML* gene members identified in the genome of *T. turgidum* and *Ae. tauschii*, respectively (Additional file 5: TableS3). Collinearity diagrams among *TaCaM/CML* gene members were further analyzed using MCscanX sofeware. Among 248 *TaCaM/CML* gene members in wheat, 230 and 222 gene members were found with 1–4 paralogous genes in *T. turgidum* and *Ae. Tauschii*, respectively (Figure 3; Additional file 6: TableS4).

### Cis*-acting elements in the promoters of the* TaCaM/CMLs *genes*

Phytohormone and stress responses *cis*-acting elements in the promoter regions of the 321 *TaCaM/CMLs* transcripts were predicted: auxin responsive element (AuxRR-core: GGTCCAT, TGA-element: AACGAC), JA-responsive element (CGTCA-motif: CGTCA), abscisic acid (ABA)-responsive element (ABRE: ACGTGG/TC), dehydration-responsive element/C-repeat element (DRE/CRT: A/GCCGAC), environmental signal response element (G-box: CACGTG), low-temperature responsive element (LTR: CCGAAA), WRKY binding site (W-box: T/CTGACC/T), and sulfur-responsive element (SURE: GAGAC).

**Figure 4.**
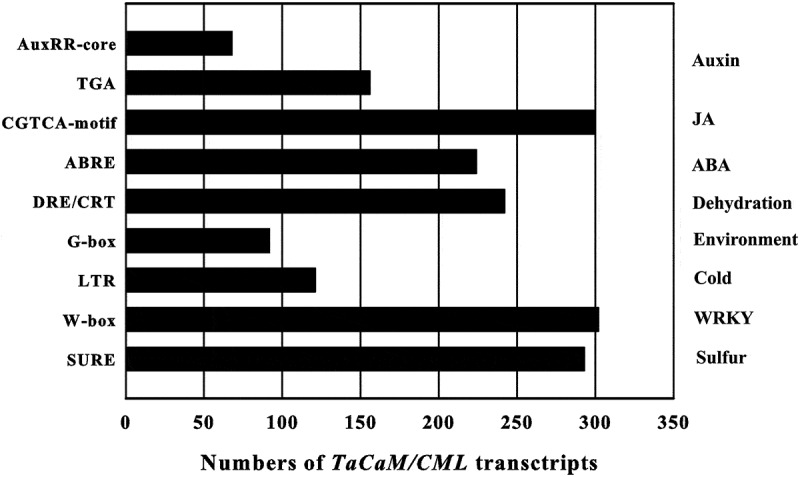
Numbers of *TaCaM/CML* transcripts containing various *cis*-acting elements. AuxRR-core and TGA element: auxin responsive element, CGTCA-motif: JA-responsive element, ABRE: abscisic acid (ABA)-responsive element, DRE/CRT: dehydration-responsive element/C-repeat element, G-box: environmental signal response element, LTR: low-temperature responsive element, W-box: WRKY binding site, SURE: sulfur-responsive element.

The results showed that, among 321 *TaCaM/CML* transcripts, 68, 156, 300, 224, 242, 92, 121, 302, and 293 of *TaCaM/CML* transcripts have AuxRR-core, TGA-element, CGTCA-motif, ABRE, DRE/CRT, G-box, LTR, W-box, and SURE in their promoters, respectively (Figure 4), while 1, 18, 59, 95, 89, 42, 15, and 2 transcripts have 9, 8, 7, 6, 5, 4, 3, 2 typies of *cis*-acting elements in their promoters, respectively. (Additional file 7: Table S5).

### *The spatiotemporal expression patterns of* TaCaM/CML *genes*

**Figure 5.**
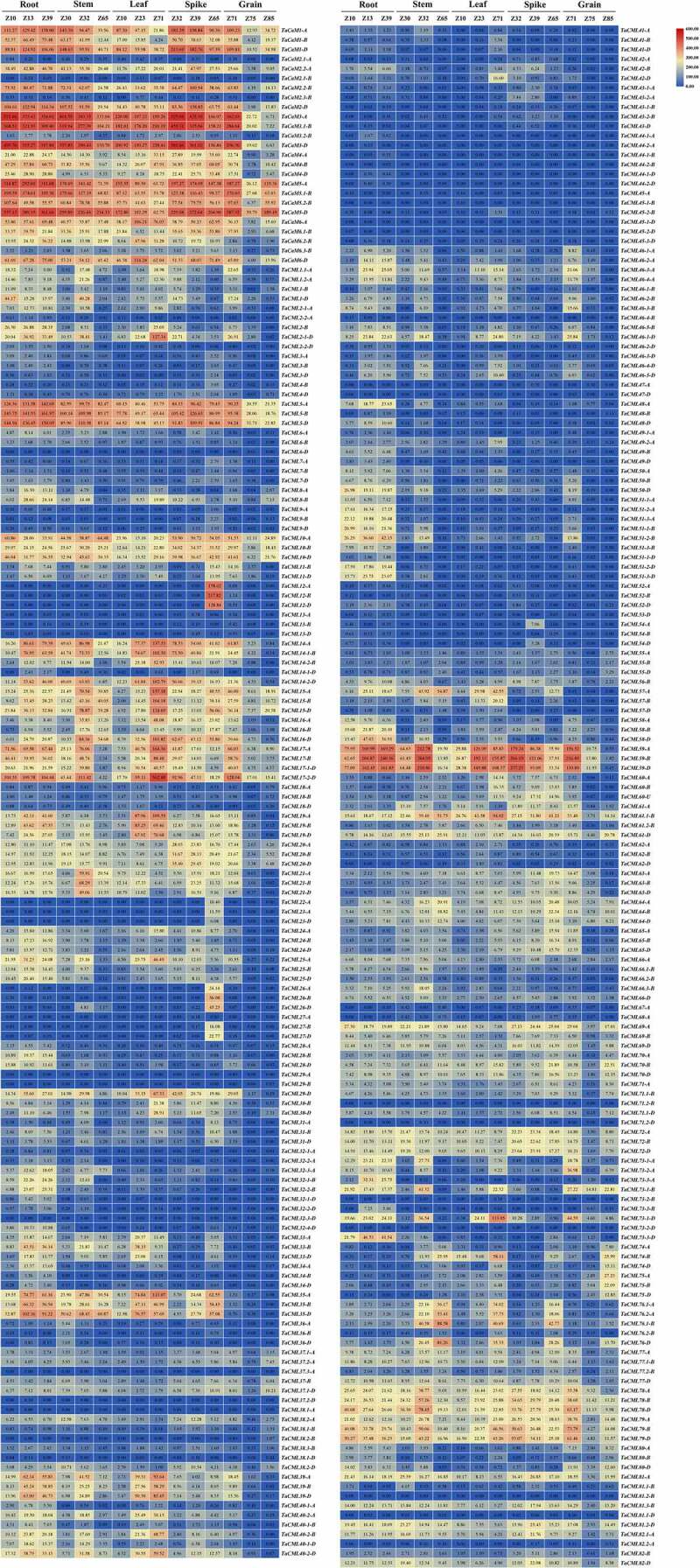
Spatiotemporal expression patterns of *TaCaM/CML* genes. The heat map illustrates the spatiotemporal expression patterns of *TaCaM/CML* genes in five tissues (roots, leaves, stems, spikes, and grain) in three developmental stages. A Zadoks scale represents the developmental stages. Different colors correspond to log_2_-transformed values. Blue or red indicates lower or higher expression levels of each transcript in each sample, respectively.

To detect the spatiotemporal expression patterns of the *TaCaM/TaCML* genes, the gene expression TPM values of 321 *TaCaM/CML* transcripts in three developmental stages of five different tissues (including the root, stem, leaf, spike, and grain) were calculated by exploiting the previously reported RNA-Seq data.^48^ The heatmap of the expression patterns of the *TaCaM/TaCML* transcripts were prepared using the TPM values (Figure 5). Most of the *TaCaM/TaCML* genes were detected in at least one tissue, except for *TaCML29-A, 32-3-D, 38.1-A, 45-1-D, 73-2-D*, and *81.2-B*, which can not be detected in any tissue. Several genes are expressed at almost one time point in specific tissue; for example, the expressions of *TaCML12-A/B/D, TaCML22-A, TaCML23-A/D, TaCML26-A/B/D*, and *TaCML27-B/D* can be detected mainly in the late stage (Z65) during spike development, implying their specific functions. Generally, most gene members from the same cluster showed similar but not exactly the same expression patterns, implying their redundant and partially differentiated functions. However, there are some transcript variations or gene duplications showed different expression levels; for example, *TaCaM2.2-A/2.2-B* was highly expressed in all detected tissues, while the expressions of *TaCaM2.1-A/2.1-B/2.3-B* was lower (Figure 5).

### *Expression patterns of* TaCML *genes under abiotic stress*

**Figure 6.**
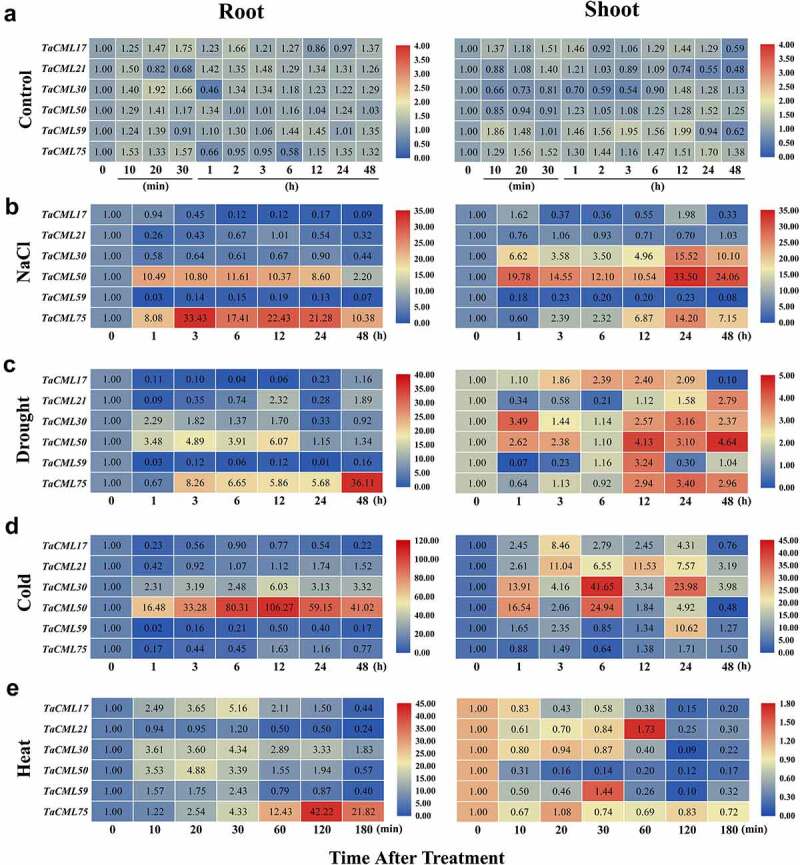
Relative expression levels of *TaCML* genes after abiotic treatment. The heatmap shows the relative expression patterns of *TaCML* genes in the roots and shoots after NaCl (a), drought (b), cold (c), and heat (d) treatments using qRT-PCR analysis. Different colors correspond to relative expression values. Blue or red indicates lower or higher relative abundance of each transcript in each sample, respectively.

In order to study whether wheat *CaMs/CMLs* are involved in the plant responses to abiotic stresses, 6 *CML* gene clusters – *TaCML17, 21, 30, 50, 59*, and *75* – were selected to analyze their expression patterns in the root and shoot of 10-day seedlings after NaCl, polyethylene glycol (PEG), cold, and heat treatment using quantitative real-time PCR (qRT-PCR) methods. In control seedlings, the expression levels of 6 *CML* genes showed no significant changement in root or shoot (Figure 6a). Under NaCl stress, the expressions of *TaCML50* and *75* in root, and *TaCML30, 50* and *75* in shoot increased significantly; while the expressions of *TaCML17, 21* and *59* in root, and *TaCML17* and *59* in shoot decreased (Figure 6b). In response to PEG treatment, the expressions of *TaCML30, 50* and *75* in root, and *TaCML17, 30, 50* and *75* in shoot were markedly enhanced; and the expressions of *TaCML17* and *59* in root were inhibited; while the expressions of *TaCML21* in root, and *TaCML21* and *59* in shoot were inhibited at early stage and up-regulated afterward (Figure 6c). In the cold treatment assay, the expressions of *TaCML30* and *50* in root, and *TaCML17, 21, 30, 50* and *59* in shoot were significantly up-regulated; while the expressions of *TaCML17, 59* and *75* in root were down-regulated (Figure 6d). In the heat treatment group, the expressions of *TaCML17, 30, 50, 59* and *75* in root, and *TaCML21* in shoot increased; while the expressions of *TaCML17, 30, 50* and *59* in shoot decreased (Figure 6e).

## Discussion

In this study, 18 *TaCaM* members from 6 gene clusters and 230 *TaCML* members from 82 gene clusters were identified in the wheat genome, which can be classified into 7 subfamilies. It can be found that the size of *CaM/CML* family in wheat is larger than most plant species, including *Arabidopsis* (7 *CaMs* and 50 *CMLs*),^4^ rice (5 *CaMs* and 32 *CMLs*),^23^ tomato (6 *CaMs* and 52 *CML* genes),^24^ and lotus (7 *CaMs* and 19 *CMLs*).^26^ Considering that wheat is heterohexaploid, the size of *CaM/CML* family in wheat is similar to cabbage (79 *CML* genes),^27^ apple (7 *CaMs* and 83 *CMLs*),^49^ *T. turgidum* (AABB genome, 152 *CaM/CMLs*), and *Ae. Tauschii* (DD genome, 88 *CaM/CMLs*) (Additional file 5: TableS3). It can be found that the ancestral species contained a large numbers of *CaM/CML* genes, and most of *TaCaM/CML* gene members were found with orthologous genes in *T. turgidum* and *Ae. Tauschii*, as well as a few numbers of *TaCaM/CMLs’* orthologous genes were not found (Figure 3; Additional file 6: TableS4). It indicated that the high numbers of *TaCaM/CML* genes may be due to the whole-genome duplications during chromosome polyploidization, and gene duplication after polyploidization event.

Most *TaCaM/CML* genes from the same cluster had similar gene structures and protein motifs, as well as located in the same homoeologous chromosome group, except for *TaCML77* and *TaCML82*, in which *TaCML77-A* and *TaCML82-A* were located in 5A, while *TaCML77-B* and *TaCML82-B* were located in 4B, and *TaCML77-D* and *TaCML82-D* were located in 4D (Figure 2). This may be due to the 4AL/5AL reciprocal translocation during the structural evolution of wheat chromosomes.^50^

In previous work, a southern-blot analysis used a probe derived from the 3ʹ-UTR of *TaCaM1-3*, suggested *CaM* genes from cluster 1 were located only on 3AS and 3BS, but not on 3DS.^29^ However, they were located in all three homoeologous-group 3 chromosomes (3A, 3B, and 3D) in our analysis, which may be because the probe in the southern-blot analysis was not effective enough to detect *TaCaM1* gene member located in chromosome 3D.

The differential spatiotemporal expression patterns of *TaCaMs/CMLs* may provide important clues for exploring their functions in growth and development of wheat in the future. In this study, most of the *TaCaM/CML* genes can be detected in at least one tissue (Figure 5), implying wide involvement of *TaCaM/CML* genes in wheat growth and development. However, *TaCML29-A, 32-3-D, 38.1-A, 45-1-D, 73-2-D*, and *81.2-B* were not expressed in any tissue (Figure 5), suggesting these genes may be related to other biological process, such as abiotic or biotic tolerance, or due to homoeologous gene silencing.^51^

During growth and development, wheat is often affected by abiotic stresses, which causes great losses to wheat production. It suggested that the losses in wheat yields caused by abiotic stresses such as salinity, drought, and heat more than biotic influences.^52^ Therefore, it is indispensable to understand the complex network of wheat responses to abiotic stresses for improving wheat yields. Increasing evidence shows that *CaMs/CMLs* in other plant species are involved in responses to abiotic stresses, including heat, cold, drought, and salinity.^15,17–22^ In *Arabidopsis*, it has been showed that *AtCaM1, 3*, and *4*, as well as *AtCML9, 18, 24, 37*, and *42* were involved in resistance to abiotic stresses.^15,22,53–58^ Additionally, *OsMSR2, OsCML4*, and *OsCaM1-1* in rice,^18–20,59^ *GmCaM4* in soybean,^21^ *MtCML40* in alfalfa,^60^ and *ShCML44* in wild tomato^17^ were related with tolerance to abiotic stresses in plants. In this study, high numbers of *cis*-acting elements related to plant hormones and stress responses can be observed in the promoters of *TaCaM/CMLs* (Figure 4; Additional file 7: Table S5), implying the potential roles in plant growth and resistence to abiotic stresses. It also showed that *TaCML17, 21, 30, 50, 59*, and *75* can be up-regulated significantly in at least one stress treatment (Figure 6), implying wide involvement of *TaCaM/CML* genes in resistance to abiotic stresses, providing important clues for future function studies of *TaCaMs/CMLs* in wheat.

## Supplementary Material

Supplemental Material

## Data Availability

All data generated or analysed during this study were included in this published article and the additional files.

## Abbreviations

Ca^2+^: Calcium; [Ca^2+^]_cyt_: Ca^2+^ concentrations; CaM: Calmodulin; CBK3: CaM-binding protein kinase 3; CBL: Calcineurin B-like; CDPKs/CPKs: Ca^2+^-dependent protein kinases; CML: Calmodulin-like; GSNOR: S-nitrosoglutathione reductase; HSF: Heat shock transcription factor; ML: Maximum likelihood; MW: Molecular weight; NO: Nitric oxide; qRT-PCR: pI: Isoelectric point; PP7: Protein phosphatase 7; Quantitative real-time PCR; TPM: Transcripts per million.
