# Supplementary figures and images for: Genome-wide identification and expression analysis of calmodulin and calmodulin-like genes in wheat (Triticum aestivum L.)
Source: Plant Signal Behav. 2022 Jan 17;17(1):2013646. doi: 10.1080/15592324.2021.2013646 (PMC8959510; doi:10.1080/15592324.2021.2013646)

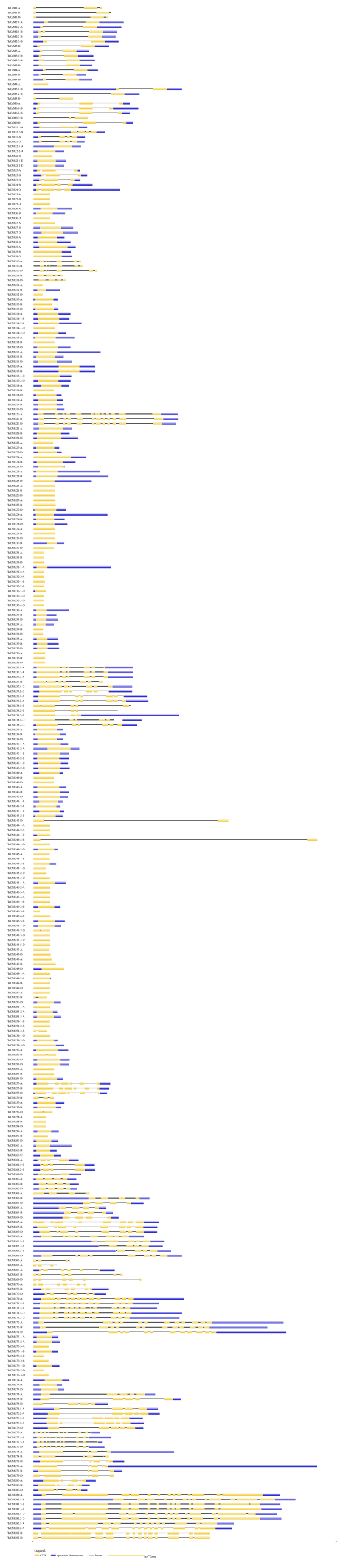

Supplement: Supplemental Material [file KPSB_A_2013646_SM9055.zip › Additional file 2 Figure S1-R.tif]
